# Mesenchymal Stem Cell Therapy for Inflammatory Skin Diseases: Clinical Potential and Mode of Action

**DOI:** 10.3390/ijms18020244

**Published:** 2017-01-25

**Authors:** Tae-Hoon Shin, Hyung-Sik Kim, Soon Won Choi, Kyung-Sun Kang

**Affiliations:** 1 Adult Stem Cell Research Center, College of Veterinary Medicine, Seoul National University, Seoul 08826, Korea; thshin1125@gmail.com (T.-H.S.); nomnoos@gmail.com (S.W.C.); 2 Research Institute for Veterinary Science, College of Veterinary Medicine, Seoul National University, Seoul 08826, Korea; 3 Biomedical Research Institute, Pusan National University Hospital, Busan 49241, Korea; hskimcell@pusan.ac.kr; 4 Department of Medical Science, School of Medicine, Pusan National University, Busan 49241, Korea

**Keywords:** mesenchymal stem cells, stem cell therapy, immunomodulation, inflammatory skin diseases, atopic dermatitis, psoriasis

## Abstract

Inflammatory skin disorders that cause serious deterioration of the quality of life have become one of the major public concerns. Despite their significance, there is no fundamental cure to date. Mesenchymal stem cells (MSCs) possess unique immunomodulatory properties which make them a promising tool for the treatment of various inflammatory diseases. Our recent preclinical and clinical studies have shown that MSCs can be successfully used for the treatment of atopic dermatitis (AD), one of the major inflammatory skin diseases. This observation along with similar reports from other groups revealed the efficacy and underlying mechanisms of MSCs in inflammatory dermatosis. In addition, it has been proposed that cell priming or gene transduction can be novel strategies for the development of next-generation high-efficacy MSCs for treating inflammatory skin diseases. We discuss here existing evidence that demonstrates the regulatory properties of MSCs on immune responses under inflammatory conditions.

## 1. Introduction

Inflammatory skin diseases such as atopic dermatitis (AD) and psoriasis are considered major public issues with increasing prevalence due to the rapid industrialization of modern society [1,2]. These diseases are more frequent in industrialized countries than in developing countries with higher prevalence in urban areas compared to rural areas [1,3]. Rapid industrialization has increased production of carbon dioxide and various exhaust gases, resulting in many forms of air pollution and high-allergen environments. Allergic diseases including AD are more closely related to these environmental changes. The elevated carbon dioxide concentrations and temperatures in cities enhance the production and allergenicity of pollen, one of the well-known plant allergens [4]. In addition, diesel exhaust particles have been reported to directly induce histamine release from mast cells (MCs) with the aggravation of allergic symptoms [5]. The symptoms of these diseases can deteriorate the quality of life of patients as a result of an impaired skin barrier, itch, insomnia, and social stigma over a long period of time. However, treatments for these disorders are limited with few approved therapeutic options for patients with moderate-to-severe symptoms who are unresponsive to topical steroids or systemic immunosuppressants [6,7]. Although biologics that target a specific cytokine or mediator seem to be effective, those drugs should be treated through multiple administration to exert sufficient efficacy and their safety should be further confirmed by long-term follow-up study [8]. Mesenchymal stem cells (MSCs), the major stem cells in the field of cell therapy, have been used in the clinic for more than 10 years and proven to be safe and effective for the treatment of various intractable autoimmune and inflammatory disorders because of their distinct immunomodulatory properties [9,10,11,12]. There is also increasing interest in the therapeutic application of MSCs for inflammatory skin conditions [13]. In this review, we provide a comprehensive overview of current reports regarding MSC as an active medicinal drug that exhibits immunomodulatory activities against various inflammatory diseases, especially within the skin.

## 2. Properties of MSCs

### 2.1. Generalities

Since the first identification of MSCs from bone marrow (BM) as precursor cells of osteogenic lineage in 1970 [14], a subsequent series of investigations on these cells has made tremendous advances. MSCs are stromal-derived non-hematopoietic progenitor cells that reside in and can be expanded from various tissues of adult and neonatal origin [15,16], such as the BM [17], umbilical cord (UC) [18], umbilical cord blood (UCB) [19], adipose tissue (AT) [20], amniotic fluid [21], placenta [22], dental pulp [23] and skin [24]. MSCs have been shown to possess the multi-lineage potential to differentiate into distinct mesenchymal cell lineages, including adipogenic, osteogenic and chondrogenic lineage [25]. Moreover, several studies have reported the transdifferentiation capacities into more specialized cell types originated from other germ layers, such as neuronal cells [26] and fibroblasts [27], under certain culture conditions. Although they share fundamental characteristics, it should be considered that MSCs are heterogeneous population consisting of diverse cell types and their properties and functions thus may vary depending on the cell sources and methods of isolation, culture and manipulation of cells [16,28]. Therefore, to standardize MSCs and minimize confusion in MSC research, the International Society for Cellular Therapy (ISCT) established minimal criteria in 2006 [29]. Briefly, MSCs should adhere to plastic in culture with a fibroblast-like morphology, have multipotency of differentiation into the three major mesenchymal lineages in vitro (osteoblasts, adipocytes, and chondrocytes), and finally express specific surface markers like CD73, CD90, Sca-1 and CD105, but not the negative markers such as CD14 or CD11b, CD34 and CD45. However, several subsequent investigations have changed the concept of markers that distinguish MSCs from other cell types. Stro-1 is now commonly defined as one of the major positive markers for MSCs, whereas it is still controversial as to whether CD34 is a truly negative marker for MSCs because the loss of CD34 expression is likely not an inherent characteristic of MSCs but a phenomenon by cell culture [30]. Therefore, further studies are required to investigate MSCs phenotypes more clearly for the quality control of clinical-grade MSCs.

### 2.2. Immunological Properties of MSCs

Although it is somewhat conflicting whether MSCs are immunoprivileged or immunoevasive, MSCs are typically regarded to have hypo-immunogenicity because of their low expression level in major histocompatibility complex (MHC) class I molecules, and lack of MHC class II and co-stimulatory molecules, including CD80, CD86, and CD40, which enables MSCs to be safely used for allogeneic environment without potential risks for immune rejection [31,32]. Furthermore, even xenogeneic administration of human MSCs into mouse models has been also reported to be well-tolerated and sufficiently effective, suggesting that human MSCs can favorably exert cross-species immunosuppressive effects [33]. However, several studies reported that injected MSCs stimulate the generation of specific memory T cells and host adaptive immunity [34]. Considering that MSC therapies exhibit remarkable anti-inflammatory effects over relatively long periods of time in spite of its temporary existence in the host [35], it is expected that various factors other than immunogenicity might be involved in the complicated action of MSCs.

MSCs have been revealed to possess distinct immunomodulatory properties which induce immune tolerance in diverse inflammatory conditions [36]. Since the strong suppressive effect of BM-derived MSCs (BMSCs) on T cell proliferation was initially discovered, significant advances have been achieved in understanding precise mechanisms of immunomodulation by MSC. They exert these effects by influencing on proliferation, recruitment, function and fate of both the innate and adaptive immune cells, including T cells [37], B cells [38], dendritic cells (DCs) [39] and natural killer (NK) cells [40], which is likely mediated through direct cell-to-cell contact and paracrine fashion by secreting diverse immunoregulatory mediators [41]. These immunological properties make them as a novel approach of great promise for treating a wide range of inflammation-mediated diseases, and thus there have been broad and intensive studies elucidating the therapeutic potentials of MSCs and their underlying mechanisms in experimental animal models as well as clinical settings.

### 2.3. Therapeutic Application of MSCs

Based on in vitro results that BMSCs inhibit the proliferation of T lymphocytes [42] and in vivo data demonstrating the BMSCs-mediated prolongation of skin graft survival in a nonhuman primate model [43], Le Blanc and colleagues first revealed the therapeutic potential of intravenously (IV) injected allogeneic BMSCs in severe acute graft-versus-host disease (GvHD) [44]. Thereafter, a series of studies has been published to investigate the therapeutic efficacies and relevant mechanisms of action of MSCs. Indeed, MSCs have been extensively employed in the treatment of various autoimmune and immune-related diseases, including GvHD [45,46], systemic lupus erythematosus (SLE) [47,48], rheumatoid arthritis (RA) [49,50] and multiple sclerosis (MS) [9,51], with beneficial outcomes. Furthermore, a number of studies recently have demonstrated that MSCs can also alleviate allergic immune disorders, such as asthma [52,53], allergic rhinitis [54] and dermatitis [55,56,57], suggesting that MSCs can exert consistent anti-inflammatory effects and therapeutic efficacies against different disease-specific inflammatory status. Given that no noticeable adverse events have been reported in these studies, MSC-based cell therapies are safe and effective for treatment of severe and intractable immune-related diseases, especially for refractory patients to current first-line medications.

MSC-mediated immunomodulation has also been examined in various inflammatory skin conditions especially unresponsive to conventional therapy. In the field of dermatology, the vast majority of studies used IV administration of allogeneic BMSCs as the primary regimen to investigate immunomodulatory effects on cutaneous inflammation. Indeed, a number of clinical data support the therapeutic efficacy and safety of IV injected BMSCs in both acute and chronic GvHD with skin manifestations [58], skin symptoms in SLE [48] and severe generalized systemic sclerosis (SSc) [59]. However, there has been an increasing evidence that MSCs derived from other tissues also possess similar immunomodulatory properties and therapeutic potentials to BMSCs, and that MSCs administered via non-IV routes can attenuate skin inflammation and disease severity. Ringden et al. showed that patients with steroid-refractory acute GvHD exhibited an overall mitigation by infusion of placenta-derived MSCs [60]. One case report suggested the significant therapeutic effect of intra-BM injected allogeneic BMSCs on sclerodermatous chronic GvHD [61]. Moreover, our previous studies showed that subcutaneously (SC) administered human UCB-derived MSCs (hUCB-MSCs) can effectively ameliorate experimental mouse model of AD [56] as well as psoriasis [62]. In addition, SC injection of allogeneic hUCB-MSCs represented promising clinical efficacy and safety in patients with moderate-to-severe AD [63]. In a mouse model of allergic contact dermatitis (ACD), human gingiva-derived MSCs (hGMSCs) exhibited more pronounced therapeutic effect after local delivery directly into ear skin lesion than IV administration [64]. Although most of the studies did not explicitly specify the number of passages for infused MSCs, it is generally presumed that relatively young passages of MSCs which retain their inherent properties are mainly used. In our previous studies on AD, hUCB-MSCs at passages 5 to 7 were used in both co-culture experiments and in vivo studies, and the clinical trial was conducted using hUCB-MSCs at passage 5. Additionally, Scuderi et al. showed that SC injection of autologous AT-derived MSCs (AT-MSCs) with hyaluronic acid (HA) scaffold resulted in the significant improvement of skin symptoms in patients with SSc, and the number of passages for injected MSCs was between 2 and 3 [65].

## 3. Preclinical and Clinical Studies of MSCs in Inflammatory Dermatoses

Dermatosis is considered to have a great importance in terms of public health owing to the high prevalence and chronicity despite the non-fatal nature of the diseases. In addition, skin symptoms frequently cause disfiguration, disability, and complications with inconvenience, which can result in considerable physiopsychological burdens on patients. While MSCs therapy in dermatology was initiated as a concept of cell replacement remedy for skin defects and wound healing, accumulating evidence has recently suggested that MSC-mediated immunomodulation can be usefully applicable to the treatment of inflammatory skin conditions. In fact, the beneficial results have been observed in various preclinical models of inflammatory skin diseases, including AD, psoriasis, and scleroderma as well as autoimmune disorders that affect the skin, including GvHD and SLE (Table 1). Based on these encouraging results, many clinical trials are currently underway verifying the efficacy and safety of MSCs against these diseases (Table 2).

### 3.1. Autoimmune Skin Diseases

#### 3.1.1. Cutaneous GvHD

GvHD is a debilitating complication that might frequently develop after allogeneic BM or hematopoietic stem cell transplantation (HSCT). It is estimated that the incidence of GvHD is approximately 80% of patients with human leukocyte antigen (HLA)-mismatched GvHD and less frequently up to 50% even in HLA-matched GvHD. This disease can be classified into two forms, acute and chronic GvHD, depending on the time of onset and disease severity. The most commonly affected organs by acute GvHD are skin, liver and gastrointestinal tract, whereas manifestations by chronic GvHD can appear anywhere [78]. In acute cutaneous GvHD, erythematous maculopapular rash with pruritus often appears as the earliest symptoms, which can be a good clue for the diagnosis of GvHD. Chronic cutaneous GvHD is divided into sclerotic (sclerodermatous) or lichenoid GvHD based on the type of skin lesions and the stage of onset. Lichenoid GvHD which represents lichen planus-like cutaneous inflammation occurs at the beginning stage of chronic GvHD, and later sclerotic GvHD displaying high similarities with scleroderma appears. As the clinical and histopathological features of cutaneous GvHD are difficult to distinguish from those of various other dermatoses, careful differential diagnosis is required for proper treatment and management of the disease. The pathogenesis of cutaneous GvHD features that T cells transferred from donor recognize host HLA molecules as non-self, thereby generating the unexpected allogeneic immune responses. Importantly, it has been well established that T lymphocytes are the major effector cells of adaptive immunity and act as the primary immunocompetent players in numerous autoimmune and inflammatory disorders and transplant rejection as a result of highly specified antigen recognition and diverse effector functions. T cells are largely divided into CD4^+^ helper T (Th) cell and CD8^+^ cytotoxic T lymphocyte (CTL), both of which can differentiate into different effector subsets upon antigen-specific activation by antigen-presenting cells (APCs). Naïve Th precursor cells can differentiate into Th1, Th2 and Th17 subsets in response to the certain cytokine milieu. CD8^+^ precursor cells can also develop as different subtypes similar to Th cell counterparts. Moreover, regulatory T (Treg) cells that suppress the activation of effector cells are concomitantly generated to maintain immune homeostasis.

The diverse T cell responses play a critical role in the development of cutaneous GvHD. Recent experimental results using animal models have revealed that the pathomechanism of acute GvHD largely consists of three sequential phases. Activated host APCs under HSCT condition result in the activation, proliferation and migration of immunocompetent T cells transferred from the donor, subsequently substantial tissue damages occur mainly by CD4^+^ and CD8^+^ T cells and NK cells. Therefore, Th1-mediated CTLs have initially been regarded as major effector cells in acute GvHD, but several recent studies have reported an involvement of Th2 cells and IL-22-producing CD4^+^ T cells. By contrast, chronic GvHD has traditionally thought to be closely linked with Th2-cell responses. More recently, various studies have suggested that Th1, Th17 and Treg cells as well as B cells might contribute to the development of the disease [79].

**Figure 1:**
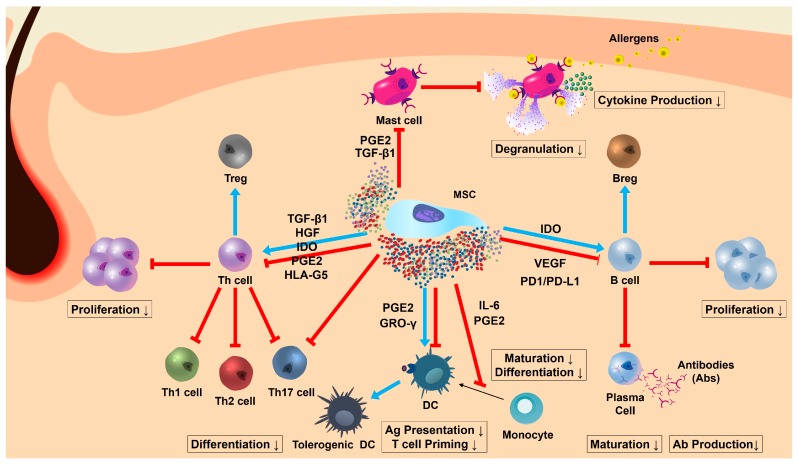
Mechanisms of MSC immunomodulation against principal inflammation-aggravating immune cells within inflammatory skin conditions. Red line: suppressive effect; Blue arrow: stimulatory effect.

Classically, early studies showed that MSCs can effectively inhibit the proliferation and differentiation of T lymphocytes through various mechanisms, including induction of cell cycle arrest [37], direct cell-to-cell contact [80], secretion of soluble mediators such as hepatocyte growth factor (HGF), transforming growth factor β1 (TGF-β1) and prostaglandin E2 (PGE2) [42,80,81] and indirect action via regulation of other immune cells like DCs or monocytes [82] (Figure 1 and Table 3). In this context, cutaneous GvHD along with skin graft rejection has been considered as the most appropriate and easily approachable target to explore the immunomodulatory effects of MSCs on T cells [43]. Indeed, adoptive transfer of BMSCs has been extensively tested in preclinical and clinical settings with beneficial achievement. IV infusion of BMSCs remarkably not only delayed the onset of GvHD and reduced skin symptoms but also prolonged skin graft survival in rat and baboon models [43,82]. In a phase II clinical study of 55 patients with steroid-resistant, severe, acute GvHD, 30 (54.5%) patients represented a complete response (CR) after IV infusion of allogenic BMSCs [12]. In another open-label clinical trial, pediatric patients with acute GvHD exhibited significant improvement by BMSCs injection, even with cutaneous GvHD (CR in 6 out of 8 patients) [77]. Transplantation of allogeneic BMSCs has been also proved to be effective for treating chronic GvHD with skin involvement [58]. Moreover, Zhou et al. reported that intra-BM injection of BMSCs resulted in gradual improvement of dermatologic symptoms in patients with sclerodermatous chronic GvHD by re-establishing Th1/Th2 balance, with an up-regulation of Th1 and a down-regulation of Th2 cytokines [61]. Based on these encouraging results from clinical trials, a cell therapy product utilizing BMSCs has been recently approved by Health Canada for the treatment of pediatric acute GvHD. Although most clinical studies so far have been profoundly focused on BMSCs, there have been many studies using MSCs from non-BM sources. Notably, allogeneic and xenogeneic AT-MSCs showed remarkable remission in a mouse model of GvHD by suppressing T cell activities [45]. Furthermore, Guo et al. demonstrated that IV injection of xenogeneic human UC-derived MSCs (hUC-MSCs) could effectively reduce the progress of murine acute GvHD and prolong the survival through indoleamine 2,3-dioxygenase (IDO)-mediated T cell inhibition [46].

#### 3.1.2. Cutaneous Lupus Erythematosus

Lupus erythematosus (LE) is a multifarious immune-mediated disease with a broad spectrum of clinical presentations provoked by impairment of self-tolerance and autoimmunity. Clinical manifestations of the disease may affect multiple tissues and organs, including the renal, neural, cardiovascular, musculoskeletal and cutaneous system with varying degrees of severity [113]. Although the mainstay of investigations has primarily focused on SLE with renal injury due to its clinical severity, there have been increased investigations demonstrating the importance of and interest in cutaneous LE (CLE). Cutaneous lesions may occur as either primary signs without systemic manifestations or as one of the comorbid symptoms associated with SLE, the most severe form of LE accompanying lethal multiorgan damages. Although the precise immunological pathogenesis of CLE has yet to be fully elucidated, complex cascades of native skin cells, such as endothelial cells and keratinocytes, and immune cells, especially Th1 cells, neutrophils and polyclonal B cells, are known to be implicated in cutaneous inflammation. Particularly, a hallmark of the CLE pathophysiology is the abnormal production of autoreactive antibodies against nuclear antigens, including RNA-binding proteins, double-stranded DNA (dsDNA) or chromatin-associated proteins, which is primarily mediated by aberrant T and B cell responses [113,114]. Moreover, disturbances in apoptotic process responsible for the clearance of dead cells cause the release of these nuclear antigens into the extracellular space, leading to the formation and deposition of immune complexes in target tissue [115].

*Fas* mutated MRL/*lpr* and NZB/W F1 mice have been widely used as experimental animal models of SLE to explore the therapeutic potential of MSCs. Indeed, IV administration of allogeneic MSCs efficiently improved multiorgan dysfunction in both MRL/*lpr* mice [47,67] and NZB/W1 F1 mice [68]. Although these studies exhibited the in vivo therapeutic effects mainly limited to nephritic exacerbations, lupus mice received MSC treatment commonly showed the down-regulated B cell activation and maturation and the reduced circulating autoantibodies. With regard to B cell function, a number of studies conducting under in vitro co-culture conditions have revealed that MSCs generally exert the suppressive effect on B cells. In fact, MSCs inhibit B cell proliferation through cell cycle arrest in the G_0_/G_1_ phase without the induction of apoptosis [38] and suppress maturation of B cells to plasma cells, antibody secretion and the expression of chemokine receptors on B cells through direct cell contact [99] or soluble mediators [98]. In addition, several reports have been proposed that T cells are needed for the MSC-mediated B cell suppression [116], whereas contradictory result have been also documented that hAT-MSCs can directly induce IL-10-secreting regulatory B (Breg) cells to reduce plasmablast formation [104] (Figure 1 and Table 3). Based on these B cell inhibition effects, it is speculated that MSCs might be effective for attenuating CLE as well as SLE.

MSC-based therapies have shown the significant clinical remission in patients with refractory SLE including skin manifestations. Liang et al. found that IV infusion of allogeneic BMSCs remarkably ameliorated the severity of nephritic and cutaneous manifestations. Among eight SLE patients with skin involvement, four patients achieved CR at one-month follow-up and skin symptoms of the other four patients also gradually faded after three months from MSC administration [48]. Conversely, Carrion et al. reported that transplantation of autologous BMSCs up-regulated circulating Treg population but failed to achieve clinical benefit in two patients with SLE [73]. Moreover, considering the result that disease relapse can occur in several patients with active and refractory SLE after 6 months from a prior allogeneic UC-MSC-driven clinical remission [74], further investigations are required to ascertain the precise efficacy of MSC injection and its long-term safety. Nonetheless, these results support the strong evidence that MSC-based therapy may produce the beneficial outcome for patients with refractory SLE, including CLE.

#### 3.1.3. Systemic Sclerosis/Scleroderma

SSc or scleroderma is a rare rheumatic disease of connective tissues accompanied by autoimmunity, vasculopathy and progressive skin fibrosis caused by excessive collagen deposition. Distinct skin lesions of the finger such as tightening and Raynaud’s phenomenon are the clinical signature of SSc, but multiorgan complications, typically pulmonary fibrosis, can be accompanied, which aggravate the disease progress to the life-threatening condition. SSc is largely classified into two subtypes according to the extent of skin involvement. The limited form of SSc affects only the skin of distal extremities and face, whereas diffused or generalized form encompasses a wide range of visceral manifestations as well as more severe skin fibrosis [117].

Although the pathogenesis of SSc is so far poorly understood, interactions between vascular, immunological and fibrotic processes are regarded as the main pathogenic factors responsible for the clinical manifestations in SSc. Firstly, environmental factors or genetic predisposition can cause endothelial damages and vascular injuries, leading to the secretion of various cytokines and mediators. In turn, these molecules can result in the impairment of innate and adaptive immune system, including the production of autoantibodies, mononuclear cell infiltration and dysregulation of cytokine production. Diverse mediators secreted from endothelial cells and activated immune cells, such as endothelin-1 (ET-1), platelet-derived growth factor (PDGF), TGF-β and IL-4, induce fibroblast activation and extracellular matrix compound (ECM) deposition [118]. Particularly, the immunological responses in patients with SSc are predominated by Th2 cells and relevant cytokines like IL-4 and IL-13, which contributes to the formation of pro-fibrotic microenvironment [119]. Because of the diversity of clinical features and complexity of pathogenesis, satisfactory therapeutic approaches for SSc are currently not available. In common with other autoimmune diseases, in spite of remarkable advances in the development of therapeutic strategies, SSc still remains an intractable disease with high morbidity and mortality [120].

Although several studies have shown the marked therapeutic potential of BMSCs and UC-MSCs in bleomycin-induced rodent SSc models, these results have been limited only to anti-fibrotic effects against the lung fibrosis and injuries but not the systemic manifestations including cutaneous scleroderma [121,122,123]. Recently, a new animal model induced by repeated application of hypochlorous acid (HClO) has been developed to examine the therapeutic effects of MSCs. As this model reflects the role of free radicals in disease progress, this model can mimic the diffuse form of human SSc more accurately. Maria et al. reported that IV injection of allogeneic BMSCs exerted significant preventive and therapeutic effects on HClO-induced murine SSc. They also showed the pleiotropic effects of MSCs, including anti-fibrotic, anti-oxidant and immunomodulatory capabilities for skin lesions as well as lung injuries [69]. In a genetic model of SSc, Akiyama and colleagues found that allogeneic BMSCs remarkably reduced hyperdermal thickness and autoantibodies, and MSCs-mediated T cell apoptosis by FAS/FAS Ligand death pathway triggered macrophages to produce TGF-β1 leading to up-regulation of Treg population (Figure 1 and Table 3). This mechanism of action was consistently confirmed in patients with SSc using allogeneic UC-MSCs [95].

Although none of these models perfectly reproduces the human disease condition, in vitro and in vivo results afford a sufficient basis for the clinical benefit of MSC transplantation in patients with SSc. The first clinical case report by Christopeit et al. represented that systemic delivery of haploidentical allogeneic BMSCs significantly improved the skin symptoms in a patient with severe generalized SSc [59]. The second result of the clinical study showed remarkable improvement in skin thickness in three out of five patients administered with BMSCs via IV route [76]. Furthermore, six SSc patients exhibited a significant remission in skin tightening without noteworthy complications after SC infusion of autologous AT-MSCs in HA solution [65], suggesting that MSC therapy might be an effective treatment tool for SSc.

#### 3.1.4. Psoriasis

Psoriasis is a common chronic autoimmune disease manifesting mainly in the skin and joint, or both, which affects approximately 2%–4% of the worldwide population [124,125]. Along with AD, psoriasis is regarded as the most important and notable inflammatory skin disorder. As its complexity beyond skin damages, patients with psoriasis have a higher risk of the systemic comorbidities, including psoriatic arthritis, metabolic syndromes, and lymphomas, which increases the disease burden [126]. Psoriasis is usually classified into five types according to the clinical manifestations. Psoriasis vulgaris, also known as plaque psoriasis, is the most common type of psoriasis characterized by clearly demarcated erythrosquamous plaques with silvery-white scales on the surface. Since it makes up approximately 80% of cases, psoriasis is primarily regarded as a dermatologic disease [127]. Psoriasis vulgaris can affect any location of the whole body surfaces, but major predilection sites are forearms, shins, around the anus and navel, the back of joints and scalp [128].

The pathogenesis of psoriasis is represented by DCs and T cell-mediated immune responses with complex cellular networks including macrophages, keratinocytes and neutrophilic granulocytes. Particularly, Th1 and Th17 subsets are widely considered the primary effector cells in psoriasis [129]. In the early stage of psoriatic skin, dermal plasmacytoid DCs produce interferon α (IFN-α) in response to complexes of host self-DNA and the epidermis-produced antimicrobial peptide LL-37 (cathelicidin), which stimulates dermal DC activation and migration into lymph nodes (LNs) [130]. Subsequently, activated dermal DC-derived IL-12 and IL-23 drive the differentiation of Th1 and Th17 cells, leading to the onset of psoriasis. In addition, tumor necrosis factor α (TNF-α) secreted from activated dermal DCs as well as other types of immune cells, including macrophages, lymphocytes, keratinocytes and endothelial cells, results in the disease aggravation [131]. Recent progress in the understanding of the pathogenic immune mechanisms of psoriasis has accelerated the development of newly emerged biologic therapies. Ustekinumab, a human monoclonal antibody that targets IL-12 and IL-23, has been reported to have more striking efficacy for the treatment of adult patients with moderate-to-severe psoriasis than anti-TNF-α drugs [132]. However, repeated administration over several months is necessary for achieving sufficient efficacy [133].

There have been several attempts to apply allogeneic or autologous HSCT to patients with severe psoriasis and comorbidities over the last two decades. However, HSCT has been reported to have a greater risk of developing secondary autoimmune diseases, such as thyroiditis, myasthenia gravis, ulcerative colitis and insulin-dependent diabetes mellitus [134,135,136,137]. Based on accumulated results, MSCs obtained from patients with psoriasis have been shown to have impaired anti-inflammatory function against Th cell subsets [138,139], suggesting that allogeneic MSC therapy is expected to be beneficial in treating psoriasis. However, few studies have shown the efficacy of MSCs as a therapeutic agent against psoriasis. Our previous preclinical study demonstrated that subcutaneous infusion of hUCB-MSCs efficiently attenuates imiquimod (IMQ)-induced psoriasis-like skin inflammation in mice by suppressing Th1, Th2 and Th17 differentiation and up-regulating Treg population. Psoriatic mice administered with MSCs exhibited the decreased skin ROS level and immune cell infiltration into the skin lesions. Moreover, the therapeutic effect of MSCs can be remarkably enhanced by the overexpression of superoxide dismutase 3 (SOD3), a strong antioxidant enzyme. In particular, SOD3-transduced as well as naïve MSCs sufficiently inhibited the differentiation of Th17 and production of IL-17 and IL-22 [62].

According to the NIH ClinicalTrials.gov database, the only clinical trial (NCT02491658) is currently underway using UC-MSCs for patients with moderate-to-severe psoriasis vulgaris. Based on early results of this clinical study, both patients infused with UC-MSCs remained relapse free of psoriasis for four to five years [71]. An additional case report from the Philippines showed that IV injection of autologous AT-MSCs significantly improved the severity of psoriasis vulgaris similar extent to methotrexate treatment for 292 days without serious adverse events [72]. Although these results are not sufficient to conclude the efficacy and safety of MSCs due to the restricted number of cases, they suggest the possibility that MSCs can be effectively used in psoriasis as reported in other autoimmune diseases.

### 3.2. Allergic Skin Diseases

#### 3.2.1. Atopic Dermatitis/Eczema

AD, also referred to as atopic eczema, is a representative inflammatory dermatopathy that features eczematous skin lesions with nasty pruritus, resulting from abnormal allergic immune responses against certain types of antigen, so-called “allergens”. It is estimated that AD affects approximately up to 20% of children [1] and 10% of adult [2]. Patients with AD, particularly in moderate-to-severe type, commonly suffer from significant sleep disturbance, depression and anxiety [140,141,142], which inflicts considerable psychological and socioeconomic burdens on patients and their families [143,144]. Clinical symptoms vary from almost subclinical to persistent disease with concomitant other allergic disorders including allergic rhinitis, asthma, and food allergy [145,146,147]. The principal peculiarities of AD pathophysiology include disruption of skin barrier function and excessive cutaneous inflammation [148]. Frequently, immunologic mechanisms of AD are characterized by dominant Th2-mediated abnormal inflammatory responses and elevated serum immunoglobulin E (IgE) and eosinophils [149,150]. Moreover, it has been recently reported that other Th cell subsets might contribute to the pathogenesis of AD [151]. It is now accepted that Th2 and Th22-driven inflammatory responses are responsible for acute AD, whereas chronic AD is mediated by Th1 responses [152]. Along with T lymphocytes, DCs are known to act as a major cellular player in the pathogenesis of AD. Several types of DCs expressing high affinity IgE receptors (FcεRI) including Langerhans cells and inflammatory dendritic epidermal cells (IDECs) are increased in AD lesion, which facilitates allergen uptake and T cell responses [153]. MCs as well as DCs express FcεRI and specific IgE-bearing MCs, called sensitized MCs, are abundant in AD lesion. Upon exposure to the specific allergen, IgE-mediated MC degranulation results in the release of preformed inflammatory mediators, such as histamine, serotonin, PG and leukotrienes, which contribute to disease exacerbation through the itch-scratch cycle and inflammatory processes through the recruitment of eosinophils and lymphocytes into the dermis [154].

Despite the development and application of intensive treatments including biologic targeted therapies like dupilumab (anti-interleukin (IL)-4 receptor), AD cannot be cured completely at present. Therefore, several studies have examined the therapeutic effects of MSCs against AD-like symptoms in experimental animal models. Na et al. demonstrated that IV injected syngeneic and allogeneic BM-derived clonal MSCs (cMSCs) can attenuate the severity of ovalbumin (OVA)-induced murine AD by reducing the infiltration of immune cells and IL-4 expression in the skin lesions and down-regulating the serum IgE level. Inhibition of T cell function via direct cell contact and nitric oxide (NO) production as well as suppression of IgE production through inhibition of class switch DNA recombination (CSR) were proposed as principal mechanisms of cMSCs [55]. Considering the results of other studies that MSCs can attenuate allergic airway inflammation by inhibiting the Th2 cell activities [86,87], it seems that this mechanism is consistent in the treatment of AD. More recently, our previous study showed that hUCB-MSCs exert significant protective and therapeutic effects against *Dermatophagoides farinae* (Df)-induced murine AD by inhibiting MC degranulation. Particularly, local SC injection of MSCs exhibited more remarkable therapeutic potential in AD mice compared with IV administration. PGE2 and TGF-β1 secreted by hUCB-MSCs are responsible for the suppression of MC degranulation and FcεRI expression, respectively [56]. Since MC is the core responder cells in allergic responses and Th2-driven inflammation, regulation of MC function including histamine release can be a potential target of MSC therapy (Figure 1 and Table 3). Moreover, in the same mouse model of AD, we also demonstrated that IV infused hAT-MSCs significantly reduced the disease severity through the suppression of B cell proliferation and maturation, and this effect was mediated by cyclooxygenase 2 (COX-2) signaling [66]. Although T cells are core effector cells in the pathogenesis of AD, B cells also exist in AD skin lesion and have a considerable role in antigen presentation to Th cells and IgE production.

Later on, the therapeutic efficacy of hUCB-MSCs was consistently confirmed by clinical trials (phase I/IIa) with moderate-to-severe AD patients [63]. The single SC administration of hUCB-MSCs showed dose-dependent clinical efficacy in AD manifestation. Namely, 6 out of 11 (55%) patients in high dose hUCB-MSCs (5 × 10^7^ cells)-treated group exhibited a 50% reduction in the Eczema Area and Severity Index (EASI50) without any noteworthy side effects. This is so far the only and first-in-class clinical study demonstrating efficacy and safety of allogeneic MSC therapy for patients with AD. However, further clinical studies with more patients and placebo group as well as mechanistic investigations verifying the mechanisms of crosstalk between MSCs and disease-related immune cells would make up the current lack of knowledge in this field.

#### 3.2.2. Allergic Contact Dermatitis

Repeated contact with specific allergens or irritants can cause epidermal inflammation and subsequent symptoms including a localized rash, which is defined as contact dermatitis. It is one of the most common dermatoses in human with a socioeconomic significance due to the intimate correlation between its prevalence and occupational attribute [155,156]. The disease is largely divided into three types according to the causative substance and the pathophysiological mechanisms: ACD, irritant contact dermatitis (ICD) and photocontact dermatitis. While ICD occurs as a result of non-immunologic direct tissue damage by toxic irritants, the pathogenesis of ACD, also referred to as contact hypersensitivity (CHS), is classified as a T cell-mediated delayed-type hypersensitivity (DTH) involved in type IV hypersensitivity reaction. During the sensitization phase after the first exposure, allergens penetrate skin barrier and conjugate with tissue proteins to become complete antigens, which activate epidermal keratinocytes to release inflammatory mediators and is endocytosed and processed by dermal DCs and Langerhans cells. Subsequently, activated DCs migrate to draining LNs (dLNs) where they prime naïve Th lymphocytes, thereby generating clonally expanded CD4^+^ and CD8^+^ effector cells as well as Treg cells. When sensitized individuals are re-exposured to the same allergen, sensitized effector T cells homing from dLNs secrete a variety of cytokines and chemokines, resulting in tissue damages and recruitment of other immune cells to the skin lesion. This phase is called the elicitation or challenge phase of ACD. MCs present in the ACD skin have been reported to participate in amplifying immune responses of elicitation phase through the secretion of TNF-α and IL-8 [156,157].

The hapten-induced murine CHS has been used as a preclinical model for human ACD. As haptens are not immunogenic by themselves but able to elicit immune responses only when binding to a protein, they have been frequently used as the most suitable allergen substitute to reproduce human disease. Lim and colleagues reported that IV infused BMSCs alleviated the murine CHS through the NO-mediated induction of activated T cell apoptosis in the dLNs [70]. In addition, Su et al. demonstrated that IV administration of human gingiva-derived MSCs (hGMSCs) significantly attenuated the symptoms of murine CHS to a similar extent in the BMSCs-injected group. CHS mice with hGMSCs treatment showed the reduced infiltration of DCs, CD8^+^ T cells, Th17 and MCs in skin lesions, and the increased Treg population at the dLNs and the contact area. GMSC-mediated suppressive effects on DCs and MCs were mediated by PGE2-dependent mechanisms [57]. Similarly, a recent study has suggested that local injection of hGMSCs can be the most effective way to attenuate murine CHS. Compared with IV injection, local administration of hGMSCs showed more significant amelioration in ear thickness and serum pro-inflammatory cytokine level through PGE2-EP3 signaling, especially when the cells injected after allergen challenge [64].

## 4. Conclusions and Future Perspectives

MSC-based cell therapy has been spotlighted as a promising approach for the treatment of inflammatory skin disorders, and relevant clinical trials are ongoing. We have highlighted the current knowledge about the interaction between MSCs and immune responses in inflammatory microenvironment of various dermatoses. Overall, MSCs seem to specifically target immune-competent cells in disease progression to attenuate their responses to inflammatory triggers. MSCs use a wide range of anti-inflammatory cytokines that suppress excessive immune responses and protect adjacent tissues. The interesting point is that the immunoregulatory activity seems to be critically altered by the microenvironmental milieu that MSCs encounter after their infusion [158]. Indeed, MSCs have been reported to exert even opposite outcomes in response to different inflammatory cues. Therefore, future studies should address the MSC responsiveness to various physiological conditions to further categorize microenvironmental cues for medicinal application of MSCs.

Another advantage of MSCs as therapeutics is their homing effect after in vivo administration. Indeed, several studies have demonstrated that MSCs can engraft into inflammatory lesions where they might exert immunomodulatory effects [66,159]. However, current reports cannot convincingly show the persistence of MSCs in vivo, especially in human bodies. Therefore, it is apparent that future work will be required to develop novel technology to track the distribution and persistence of MSCs after administration into human bodies because this knowledge of in vivo cell distribution can elucidate both safety and efficacy of MSC therapy. Moreover, these data from clinical trials might be required to optimize treatment dose, interval and frequency of administration.

More recently, researchers have tried to establish highly efficient MSCs due to their slightly limited therapeutic efficacy in clinical trials. Although satisfactory results have been observed in most of the phase I trials, MSCs have been shown only moderate efficacy in further phases per se. Therefore, novel strategies to facilitate the therapeutic benefits of MSCs are extremely required. The main potential drawback of MSC therapy is their “hit-and-run” mechanism [160], thereby prolonging their persistence in vivo and sustaining release of immunomodulatory factors have been regarded as main approaches. To ensure this end, various methods can be applied to generate highly efficient MSCs, including genetic modification through viral and non-viral modifications, bioengineering of surface receptors, and priming with biological agents [161]. Our previous reports revealed that nucleotide oligomerization domain 2 (NOD2)-primed MSCs and SOD3-overexpressed MSCs exert much higher therapeutic potential than naïve MSCs in the experimental model of AD and psoriasis, respectively [56,62]. Although these approaches have yet to be confirmed in the clinic, development of highly efficient MSCs with maximum benefits and minimum risk is expected to be a next-generation therapeutics. To do that, strict assessment and verification will be needed.

## Figures and Tables

**Table 1:** Effects of MSCs on experimental animal models of inflammatory skin conditions.

Model	Animals (Strain)	MSCs				Reference
		Source	Route	Effect	Mechanisms & Note	
AD (OVA-induced)	Mouse (BALB/c)	Mouse BM	IV	Y	T cell-suppression via NO; B cell-suppression via CSR	[55]
AD (Df-induced)	Mouse (Nc/Nga)	Human UCB	SC	Y	Inhibition of MC degranulation through PGE2 and TGF-β1	[56]
AD (Df-induced)	Mouse (Nc/Nga)	Human AT	IV	Y	B cell-suppression via COX-2	[66]
Psoriasis (IMQ-induced)	Mouse (C57BL/6)	Human UCB	SC	Y	Inhibition of various effector cells; SOD3-transduced MSC	[62]
SLE	Mouse (MRL/*lpr*)	Mouse BM	IV	Y	B cell-suppression via BAFF	[67]
SLE	Mouse (NZB/W F1)	Human UCB	IV	Y	-	[68]
SSc (HClO-induced)	Mouse (BALB/c)	Mouse BM	IV	Y	Diffuse SSc	[69]
GvHD	Mouse (B6D2F1)	Mouse AT	IV	Y	T cell-suppression	[45]
Acute GvHD	Mouse (DBA/2)	Human UC	IV	Y	T cell-suppression; TGF-β1 and IDO	[46]
Cutaneous DTH (DNFB-induced)	Mouse (C57BL/6)	Mouse BM	IV	Y	Induction of activated T cell; apoptosis in dLN	[70]
CHS	Mouse (BALB/c)	Human Gingiva	IV	Y	Suppression of DCs and MCs through PGE2	[57]
CHS	Mouse (BALB/c)	Human Gingiva/AT/BM	IV/Local	Y	PGE2-EP3 signaling	[64]

AD: atopic dermatitis; OVA: ovalbumin; Df: *Dermatophagoides farinae*; IMQ: imiquimod; SLE: systemic lupus erythematosus; SSc: systemic sclerosis; HClO: hypochlorous acid; GvHD: graft-versus-host disease; DTH: delayed type hypersensitivity; DNFB: 2, 4-dinitro-1-fluorobenzene; CHS: contact hypersensitivity; BM: bone marrow; UC: umbilical cord; UCB: umbilical cord blood; AT: adipose tissue; IV: intravenous; SC: subcutaneous; NO: nitric oxide; CSR: class switch DNA recombination; COX-2: cyclooxygenase 2; SOD3: superoxide dismutase 3; BAFF: B cell activating factor; dLN: draining lymph node; PGE2: prostaglandin E2; TGF-β1: transforming growth factor β 1; IDO: indoleamine 2, 3-dioxygenase; DCs: dendritic cells; MCs: mast cells.

**Table 2:** Clinical applications of MSCs in inflammatory skin conditions.

Disease	Type	Size	Periods	MSC Sources	Responses & Note	Reference
Moderate-to-severe AD (NCT01927005)	Phase I; Phase IIa	7 Adults; 27 Adults	4 and 12 weeks	AlloUCB	6/11 (55%) :EASI50 in high dose treated group	[63]
Moderate-to-severe Psoriasis vulgaris (NCT02491658)	Case report	2 Adults	4–5 years	AlloUC	2/2 CR; No adverse effects	[71]
Psoriasis vulgaris	Case report	1 Adult	292 days	AlloAT	Reduction in PASI	[72]
Refractory SLE (NCT00698191)	Pilot Study	15 Adults	17.2 ± 9.5 months	AlloBM	Reduction in SLEDAI; Remission of skin rash	[48]
SLE	Case report	2 Adults	14 weeks	AutoBM	No clinical effect	[73]
Active and refractory SLE (NCT01741857)	Multicenter clinical study	40 Adults	1 year	AlloUC	37/40 (92.5%) survival; 7/40 (17.5%) relapse; after 6 months	[74]
Refractory SLE (NCT00698191)	Case report	4 Adults	12–18 months	AlloBM	Recovery	[75]
Severe progressive SSc	Case report	5 Adults	4–44 months	AlloBM	2/5 (40%) improvement in MRSS	[76]
Severe progressive SSc	Case report	1 Adult	6 months	AlloBM	Marked improvement; by CD137L ligation	[59]
SSc	Case report	6 Adults	1 year	AutoAT (w/HA)	4/6: significant; 1/5: moderate; No related complications	[65]
Steroid-resistant, severe, acute GvHD	Phase II	30 Adults; 25 Children	60 months	AlloBM	30/55 (54.5%) CR; 9/55 (16.4%) PR	[12]
Sever refractory acute GVHD	Open-label	12 Children	2 years	AlloBM	7/12 (58.3%) CR; 2/12 (16.7%) PR	[77]
Acute GvHD; chronic GvHD (NCT00447460)	Phase I/II	10 Adults; 8 Adults	3 days–1 year	AlloBM	1/10 CR, 6/10 PR; 1/8 CR, 3/8 PR	[58]
Sclerodermatous chronic GvHD	Case report	4 Adults	4.6–23 months	AlloBM	Gradually improved	[61]

AD: atopic dermatitis; SLE: systemic lupus erythematosus; SSc: systemic sclerosis; GvHD: graft-versus-host disease; Allo: allogeneic; Auto: autologous; BM: bone marrow; UC: umbilical cord; UCB: umbilical cord blood; AT: adipose tissue; HA: hyaluronic acid; EASI: Eczema Area and Severity Index; PASI: Psoriasis Area and Severity Index; SLEDAI: SLE Disease Activity Index; MRSS: modified Rodnan skin thickness score; CR: complete responses; PR: partial responses.

**Table 3:** Mechanisms of MSC-mediated regulation on inflammation-exacerbating immune cells.

Cells		MSCs	Effects	Mechanism
T cells		mBMSCs [37,83]; hBMSCs [42,81,84,85]	Proliferation↓ [37,42,83,84,85]; Differentiation↓ [37]	Cell cycle arrest at G_1_ [37]; TGF-β1, HGF [42]; iNOS [83]; IDO [84]; HLA-G5 [85]
Th cells	Th1	mBMSCs [70]; hBMSCs [81]; hUCB-MSCs [62]	Differentiation↓ [37,55,62]; Cytokine production↓ [55,62,81]	PGE2 [81]
	Th2	mBMSCs [52,55,86]; mAT-MSCs [87]; hBMSCs [81]; hAM-MSCs [88,89]; hUCB-MSCs [62]	Activation↑ [81]; Differentiation↓ [62,70,88]; Cytokine production↓ [52,55,62,88]; No change [89]	TGF-β1 [52]; IFN-γ [86,87]
	Th17	mBMSCs [90,91,92]; hBMSCs [80,93]; hUCB-MSCs [62]	Th17 differentiation↓ [62,90,91,92,93]; Th17 differentiation↑ [92]; Cytokine production↓ [62,93]	IL-10 [90]; PGE2 [93]; CCR6/CCL20, CD18/CD54L [91]; COX-2 [91]
Treg cells		mBMSCs [94,95]; hBMSCs [80,81,85,96]; hUC-MSCs [97]	Treg induction↑ [80,81,93,94,95,96,97]; IL-10 production↑ [80,81,93,94,96,97]	Cell contact, PGE2, TGF-β1 [80]; IDO [97]; HLA-G5 [85]; Monocyte regulation [96]; FAS/FASL-mediated T cell apoptosis↑ [95]
B cells		mBMSCs [55,98,99]; hBMSCs [38,100,101]; hUC-MSCs [102,103]; hAT-MSCs [104]; hUCB-MSCs [66]	Proliferation↓ [38,55,66,98,103]; Proliferation↑ [102]; Differentiation↓ [38,55,66,98,103,104]; Differentiation↑ [102]; Antibody production↓ [38,98]; Antibody production↑ [102]; Chemotactic ability↓ [38]; Apoptosis↓ [87,100]; Breg induction↑ [101,104]	Cell cycle arrest at G_0_/G_1_ [38]; PGE2 [102]; VEGF [100]; IDO [101]; Unknown soluble factors [38,103]; PD-1/PD-L1 [99]; COX-2 [66]
DCs		mBMSCs [105]; hBMSCs [81,106,107,108,109,110]; hAD-MSCs [111]	Early DC maturation↓ [106,107]; Proliferation↓ [109,110]; Differentiation↓ [105]; T cell priming ability↓ [108]; Tolerogenic DC induction↑ [111]; mDC generation↓ [81]	PGE2 [106]; Cell cycle arrest at G0 state [109]; TLR4 [108]; GRO-γ [111]; IL-6 [105]
MCs		mBMSCs [112]; hUCB-MSCs [56]; hGMSCs [56]	Degranulation↓ [56,112]; Cytokine production↓ [57,112]	COX-2-dependent cell contact [112]; PGE2 [56,57]; TGF-β1 [56]

Th: helper T; Treg: regulatory T; Breg: regulatory B; DC: dendritic cell; mDC: myeloid DC; MC: mast cell; m: mouse; h: human; MSCs: mesenchymal stem cells; BMSCs: bone marrow-derived MSCs; UCB: umbilical cord blood; AM: amniotic membrane; AT: adipose tissue; GMSCs: gingiva-derived MSCs; PGE2: prostaglandin E2; TGF-β1: transforming growth factor β 1; COX-2: cyclooxygenase 2; HGF: hepatocyte growth factor; iNOS: inducible nitric oxide synthase; HLA-G5: human leukocyte antigen G5; IFN-γ: interferon gamma; IDO: indoleamine 2, 3-dioxygenase; PD-1: programmed death-1; PD-L1: PD ligand 1; VEGF: vascular endothelial growth factor; TLR: toll-like receptor; IL: interleukin; GRO: growth-regulated oncogene chemokines. The arrow of “↑” means stimulation or up-regulation; “↓“ means inhibition or down-regulation.

## Abbreviations

ACD	Allergic contact dermatitis
AD	Atopic dermatitis
APC	Antigen presenting cell
AT	Adipose tissue
BM	Bone marrow
Breg	Regulatory B lymphocyte
CD	Clusters of differentiation
CHS	Contact hypersensitivity
CLE	Cutaneous lupus erythematosus
COX-2	Cyclooxygenase 2
CTL	Cytotoxic T lymphocyte
DC	Dendritic cell
Df	*Dermatophagoides farinae*
DTH	Delayed type hypersensitivity
EASI	Eczema Area and Severity Index
FcεRI	High affinity immunoglobulin E receptor
GvHD	Graft-versus-host disease
HClO	Hypochlorous acid
HGF	Hepatocyte growth factor
HLA	Human leukocyte antigen
ICD	Irritant contact dermatitis
IDO	Indoleamine 2,3-dioxygenase
IFN	Interferon
Ig	Immunoglobulin
IL	Interleukin
IV	Intravenous or intravenously
IMQ	Imiquimod
LPS	Lipopolysaccharide
MC	Mast cell
MHC	Major histocompatibility complex
MSC	Mesenchymal stem cell
NK	Natural killer
NO	Nitric oxide
OVA	Ovalbumin
PASI	Psoriasis Area and Severity Index
PGE2	Prostaglandin E2
SC	Subcutaneous or subcutaneously
SLE	Systemic lupus erythematosus
SSc	Systemic sclerosis
TGF	Transforming growth factor
Th	T helper lymphocyte
TLR	Toll-like receptor
TNF	Tumor necrosis factor
Treg	Regulatory T lymphocyte
UC	Umbilical cord
UCB	Umbilical cord blood
